# Myocardial Infarction: The Protective Role of MiRNAs in Myocardium Pathology

**DOI:** 10.3389/fcvm.2021.631817

**Published:** 2021-03-05

**Authors:** Wei Wang, Hao Zheng

**Affiliations:** ^1^Graduate School of Bengbu Medical College, Bengbu, China; ^2^Department of Cardiovascular Medicine, Zhejiang Provincial People's Hospital, Hangzhou, China

**Keywords:** MI, apoptosis, hypertrophy, fibrosis, miRNA

## Abstract

Cardiovascular diseases have been regarded as the leading cause of death around the world, with myocardial infarction (MI) being the most severe form. MI leads to myocardial apoptosis, cardiomyocyte fibrosis, and cardiomyocyte hypertrophy, ultimately leading to heart failure, and death. Micro RNAs (miRNAs) participate in the genesis and progression of myocardial pathology after MI by playing an important regulatory role. This review aims to summarize all available knowledge on the role of miRNAs in the myocardial pathological process after MI to uncover potential major target pathways. In addition, the main therapeutic methods and their latest progress are also reviewed. miRNAs can regulate the main signaling pathways as well as pathological processes. Thus, they have the potential to induce therapeutic effects. Hence, the combination of miRNAs with recently developed exosome nanocomplexes may represent the future direction of therapeutics.

## Introduction

Myocardial infarction (MI) is defined as the death of myocardial cells due to prolonged ischemia and is the most serious manifestation of coronary artery disease (1). However, MI also results in cardiac remodeling, including myocardial fibrosis and cardiac hypertrophy (2). The pathological changes induced by MI can lead to heart failure, cardiac rupture, sudden death, and other adverse events (3). Antithrombotic agents, percutaneous coronary intervention, and bypass surgery are usually applied to treat patients after MI (4, 5). Nonetheless, these approaches only reduce the severity of the coronary artery disease rather than saving the ischemic myocardium and preventing the development of adverse tissue remodeling (6, 7). Therefore, novel therapeutic strategies to reduce myocardial cell death, inhibit adverse remodeling, and/or stimulate heart regeneration are highly needed.

**Figure 1:**
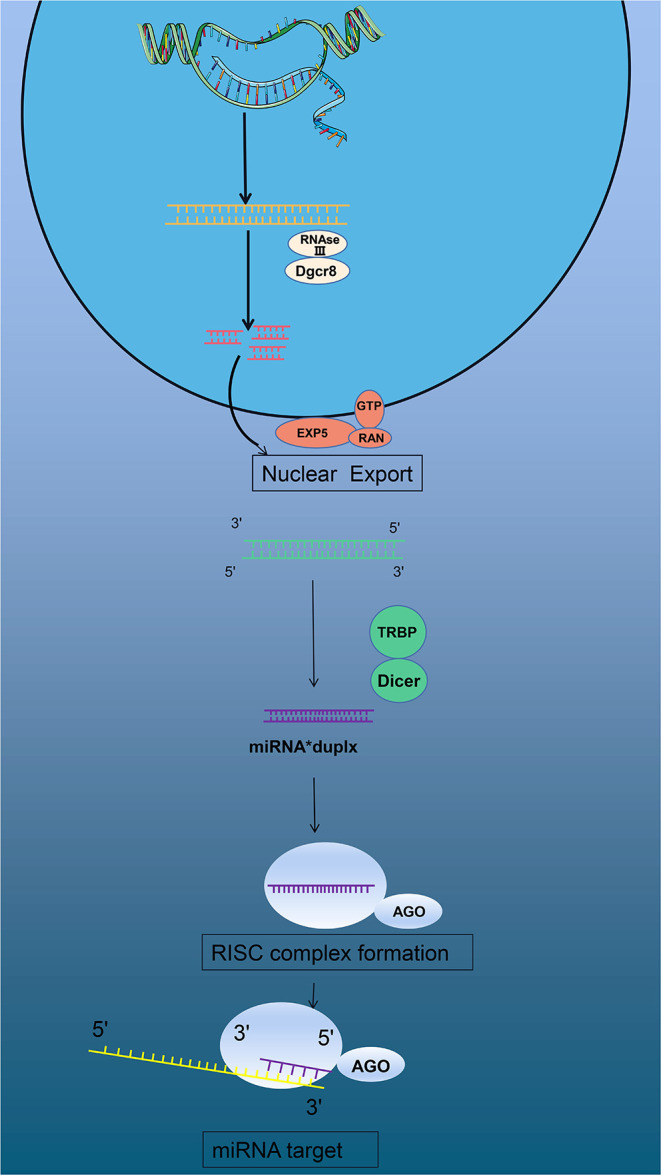
miRNAs form and function.

Micro RNAs (miRNAs) are also involved in differential gene expression in the pathophysiology of MI (5, 8). miRNAs originating from DNA sequences are transcribed by RNA polymerase II in the nucleus to form primary products: primary miRNA (pri-miRNA). Pri-miRNA is generally larger than 1000 bPs and is a double-stranded RNA, similar to a long hairpin, consisting of multiple nucleotide fragments. In the nuclear region, endonuclease Drosha (RNAse III) and cofactor Dgcr8 constitute a unique structure-microprocessor. This complex could precisely cut pri-miRNA and degrade it into a 65 bPs secondary product: miRNA precursor (pre-miRNA) (9). Subsequently, these new pre-miRNAs are transported into the cytoplasm through transport complexes that are made of export protein 5 (EXP5), RAN, GTP, and pre-miRNAs (10, 11). Once the complex passes through the nuclear membrane, the RNAse protein (Dicer) clefts the pre-miRNA into about 19-25 bPs miRNAs, and TAR RNA-binding protein (TRBP or PACT) changes the product into double-stranded miRNAs (12, 13). New double-stranded miRNAs are loaded into a specific AGO protein to form a pre-RNA-induced silencing complex (pre-RISC). As one strand of the double helix in the pre-RISC degrades, it is immediately modified to become a mature RISC (14). The complex binds to the 3'UTR region of the target mRNA, resulting in degradation or inhibition of the target mRNA (15) (Figure 1).

In the last century, the use of stem cells was thought to be a promising strategy for the treatment of MI (16). As research continues, the exact mechanism of cardiac repair by transplanted cells remains controversial and has yielded inconsistent results. Two main hypotheses exist: (a) direct cardiogenesis/angiogenic differentiation, and (b) indirect stimulation of the regenerative process through paracrine (17, 18). Leda et al. successfully reprogrammed fibroblasts directly into functional cardiomyocytes (19). But low conversion rates and a complex and expensive process have stalled the technology (20). The therapeutic role of exosomes has long been thought to be useful in the treatment of heart injury (21). In combination with nanomaterials, cell transformation is greatly improved (22).

**Figure 2:**
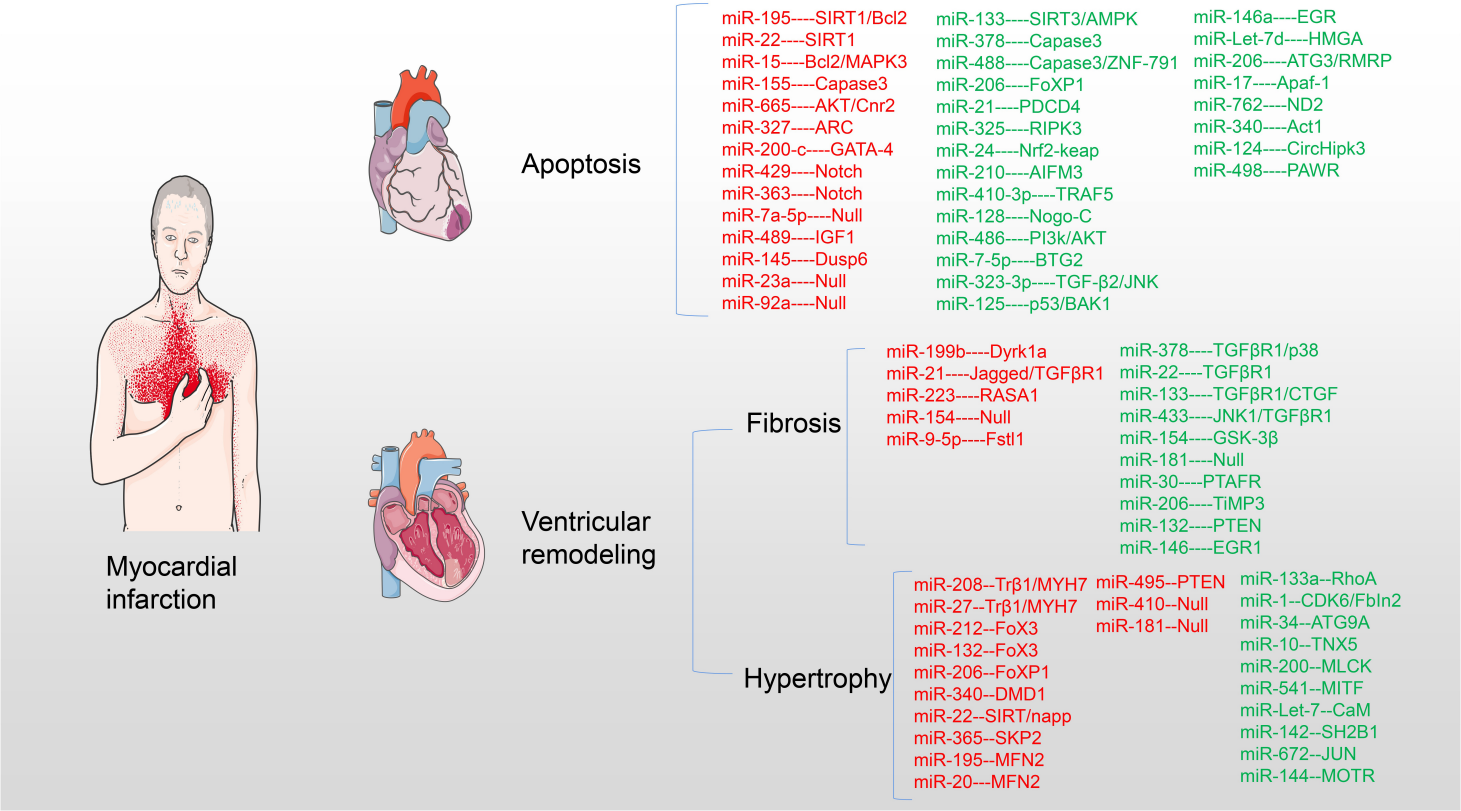
miRNAs target myocardium in pathological process after MI. miRNAs are involved in myocardial cell apoptosis, myocardial fibrosis, and myocardial hypertrophy acting on myocardial targets after MI. Upregulation of red miRNAs promoted the development of pathology, while upregulation of green miRNAs inhibited or even reversed the pathological process.

Small non-coding micro RNAs (miRNAs) participate in the pathogenesis and development of myocardial pathology after MI and play an important regulatory role. This study provides a comprehensive overview of miRNAs affecting the pathology after MI and acting on potential targets and access mechanisms. Furthermore, the present therapeutic methods of saving infarct myocardium and latest research progress are summarized. In particular, the challenges and clinical prospects of using miRNA targets for myocardial regenerative therapy are discussed (Table 1 and Figure 2).

**Table 1:** Micro RNAs targets and functions in cardiomyocyte apoptosis.

	**Functions**	**miRNA**	**Up/down**	**Targets**	**Reference**
Cardiomyocyte apoptosis	Pro-apoptosis	195	Up	SIRT1	PMID:21622680
		195	Up	Bcl2	PMID:27489501
		22	Up	SIRT1	PMID:27174562
		15	Up	Bcl2	PMID:28814571
		155	Up	Capase3	PMID:31191799
		665	Up	AKT/Cnr2	PMID:31026731
		206	Down	ATG3	PMID:29880830
		206	Down	ATG3	PMID:30551524
		17	Down	Apaf-1	PMID:26265044
		762	Down	ND2	PMID:31235686
		340	Down	Act1	PMID:30989715
		124	Down	CircHipk3	PMID:31799682
		498	Down	PAWR	PMID:32767028
	Anti-apoptosis	133	Up	SIRT3	PMID:32575874
		378	Up	Capase3	PMID:22119805
		488	Up	Capase3	PMID:31210328
		206	Up	FoXP1	PMID:26333362
		21	Up	PDCD4	PMID:29674977
		325	Up	RIPK3	PMID:31248365
		24	Up	–	PMID:25352422
		210	Up	AIFM3	PMID:32513270
		410-3p	Up	TRAF5	PMID:31696495
		182	Up	Nogo-C	PMID:27763637
		24-3p	Up	Nrf2	PMID:30622671
		486	Up	PI3k/AKT	PMID:30844685
		7a-5p	Up	BTG2	PMID:32945347
		323-3p	Up	TGF-β2	PMID:32633390
		125-b	Up	p53/BAK1	PMID:30613290
		146a	Up	EGR	PMID:30362610
		let-7d	Up	HMGA	PMID:30934671
		23a/92a	Down	–	PMID:28662151
		145	Down	Dusp6	PMID:30883744
		489	Down	IGF1	PMID:32880387
		7a-5p	Down	–	PMID:33029099
		363	Down	Notch	PMID:28402919
		429	Down	Notch	PMID:27082497
		200-c	Down	GATA-4	PMID:28440427
		327	Down	ARC	PMID:31587299

*miRNAs are divided into pro-apoptosis and anti-apoptosis according to functions. The pro-apoptosis of main targets include SIRT, Bcl, caspase, and ATG3 and the anti-apoptosis of main targets include SIRT, caspase, and AKT*.

## Cardiomyocyte Apoptosis

Apoptosis is a type of programmed cell death promoted by extrinsic and intrinsic pathways through the activation of death receptors and mitochondria, respectively (23–25). The transduction of apoptosis signals is mediated by several pro- and anti-apoptotic factors, including the caspase family, the B cell lymphoma 2 (Bcl-2) family, cytochrome c, and inhibitor of apoptosis proteins (IAP) (26). miRNAs play an important role in myocardial cell apoptosis and heart protection after MI (27–30). Two studies indicated that upregulation of miR-195 and miR-15 in ischemic cardiomyocytes of rats promotes ischemic apoptosis by targeting Bcl-2 (31, 32). In turn, miR-17 can support apoptosis via apoptotic protease activating factor 1 (Apaf-1) which facilitates the formation of apoptosomes containing cytochrome c and deoxyadenosine triphosphate (dATP) (33). However, a previous study suggested that miR-327 inhibits cardiomyocyte apoptosis *in vitro* and *in vivo* in rats by targeting the apoptosis repressor with the caspase recruitment domain (ARC) (34). In addition, miR-378 was reported to inhibit caspase-3 expression and attenuate ischemic injury in cardiomyocytes (35), whereas miR-155 did not (36). Overexpression of miRNA-488-3p markedly downregulated the levels of caspase-3 in MI (37). Furthermore, a study revealed that autophagy-related 3 (ATG13) interacts with the fas-associated protein with the death domain to promote the activation of caspase-8 and cell apoptosis (38). ATG13 is also a target of miR-206 and activates the apoptotic factor forkhead box protein 1 (FOXP1) (39, 40). Upregulation of miR-133 can inhibit cardiomyocyte apoptosis, inflammation, and oxidative stress through a mechanism that may be related to the regulation of the SIRT3/AMPK pathway (41). miR-133 is a heart- and muscle-enriched miRNA (42). Sirtuin 1 (Sirt1) also has anti-apoptotic effects that are associated with a reduction in the levels of reactive oxygen species (ROS) (43). In turn, silencing of Sirt1 abolishes the protective effects of miR-22 on hypoxia/reoxygenation-induced mitochondrial dysfunction and cell injury in cardiomyocytes (44). miRNAs also directly suppress the expression of the programmed cell death (PDCD) family, active IAPs, and accelerates cell transcription to inhibit cell apoptosis after MI (45–47). Recently, receptor interacting protein kinase 3 (RIPK3), apoptosis-inducing factor 3 (AIFM3), and tumor necrosis factor receptor-associated factor 5 (TRAF5) were confirmed to be suppressed by miRNAs (48, 49).

Myocardial cells suffer hypoxic damage when MI occurs (50). Two studies confirmed that the activation of protein kinase B (AKT) (51), as well as the ectopic expression of Notch1 (52), inhibited hypoxia-induced apoptosis in culturing human cardiomyocytes under hypoxic conditions (53). Nogo-C is an endoplasmic reticulum protein ubiquitously expressed in tissues including in the heart, which is upregulated in mouse hearts after MI and in cardiomyocytes upon hypoxic treatments (54). Furthermore, knock-down of endogenous NADH dehydrogenase 2 (ND2) significantly decreases intracellular ATP levels and mitochondrial complex-I enzyme activity, whereas it increases ROS levels and apoptotic cell death in cardiomyocytes (55). A study used a H9C2 cardiomyocyte cell line to perform *in vitro* stimulated ischemia/reperfusion (SI/R) and found a novel function of miR-24-3p in protecting cardiomyocytes from oxidative injury by the activation of the Nrf2/Keap1 pathway (56). Moreover, overexpression of miR-323-3p was also found to reduce oxidative stress and apoptosis of cardiomyocytes via the regulation of the TGF-β2/JNK pathway (57). Additionally, there are still conflicting results regarding miR-7a-5p's protective role on cardiomyocytes upon hypoxic injury (50, 58).

Moreover, upregulation of miR-340-5p suppresses apoptosis and oxidative stress induced by hypoxia/reoxygenation in H9C2 cells by inhibiting the NF-κB activator 1 (Act1) (59). Lastly, a study suggested that bone marrow mesenchymal stem cell (BM-MSC)-derived vascular endothelial growth factor attenuates cardiac apoptosis via regulation of cardiac miRNA-23a and miRNA-92a in a rat model of multiple sclerosis (60). miRNAs from BM-MSCs can interact with myocardial cells through exosomes (61). Interestingly, exosomes originating from adipose-derived stem cells can also attenuate myocardial damage triggered by acute MI via downregulation of early growth response factor 1 (Egr1) (62).

In summary, to date, more miRNAs with anti-apoptotic activity have been reported than those with pro-apoptotic effects, most of which act on classical pathways such as Bcl-2, caspase, AKT, SIRT, and apoptotic factors.

## Myocardial Fibrosis

Myocardial fibrosis is an important feature of most cardiac pathological conditions (63), characterized by alteration of the extracellular matrix (64). Currently, five types of collagen are known to be expressed in the myocardium, among which fibrillar collagen type I (85%) and type III (11%) are commonly expressed in the cardiac extracellular matrix. And, the basement membrane of myocytes and the pericellular space are rarely composed of collagen type IV and V (65). Additionally, fibrillar collagen type VI is related to the adhesion of cellular fibers (66). An MI model was established in SD rats using the LAD ligation method and the study found transforming growth factor-β 1 (TGF-β1) induces the upregulation of miR-21 and downregulation of Jagged1 in cardiac fibroblasts (CFs), which are activated by MI, thereby inducing myofibroblast transformation (67). Additionally, decreased levels of antizyme inhibitor (AZIN1) activate TGF-β1. Furthermore, downregulation of c-Jun N-terminal kinase 1 (JNK1) results in the activation of the extracellular signal-regulated kinase and p38 kinase, leading to Smad3 activation and ultimately cardiac fibrosis (68). miR-133a expression in the infarct border zone of myocardial tissue was found to be significantly decreased after MI. And, the upregulation of miRNA-133a in the myocardial tissue of rats with MI remarkably improved cardiac function and reduced collagen volume fraction (69). Furthermore, the mRNA and protein levels of TGF-β1, connective tissue growth factor, collagen I and III, and α-smooth muscle actin (α-SMA) in myocardial tissue were obviously decreased after miRNA-133a upregulation (70). A study also suggested that miR-223 mimics could enhance cell proliferation and migration, collagen I and III, and α-SMA expression in CFs, which could be mediated via mitogen-activated protein kinase kinase (MEK) 1/2, ERK1/2, and AKT phosphorylation (71). miR-154 has similar functions via glycogen synthase kinase 3 beta (GSK-3β) including reducing the heart and cardiomyocyte size, cardiac fibrosis, lowering the expression of atrial (ANP) and B-type natriuretic peptides (BNP), and of profibrotic markers (72), whereas it increases the expression of p15 (a miR-154 target and cell cycle inhibitor) (73). Furthermore, miR-378 and miR-181a are secreted by cardiomyocytes to act as inhibitors of excessive cardiac fibrosis through a paracrine mechanism (74, 75). Upregulation of miR-132 or phosphatase and tensin homolog (PTEN) silencing activate the PI3K/Akt pathway, thereby repressing cardiomyocyte apoptosis and cardiac fibrosis (76). An earlier study showed that an injection of high mobility group box 1 (HMGB1) into the heart of mice, immediately after MI, had the potential to improve cardiac regeneration and prevent remodeling (77). Recently, a study on CFs isolated from mice hearts upon angiotensin II (Ang II)-induced cardiac fibrosis post-MI revealed that miR-30b-5p and miR-22-3p were downregulated, whereas the platelet activating factor receptor (PTAFR) was upregulated [X. S. (78)]. In addition, miRNAs can directly inhibit myocardial fibrosis and even reverse ventricular remodeling (79, 80). Cardiac CITED4 (CBP/p300-interacting transactivators with E [glutamic acid]/D [aspartic acid]-rich-carboxylterminal domain 4) is sufficient to cause physiological hypertrophy and mitigate adverse ventricular remodeling after MI (81). Although few studies specifically investigated myocardial fibrosis, TGF-β1 is clearly a direct or indirect target underlying this process. Upstream targets of PTAFR and CITED4 have recently been found to be worthy of further exploration (Table 2).

**Table 2:** Micro RNAs targets and functions in myocardial fibrosis.

	**Functions**	**miRNA**	**Up/down**	**Targets**	**Reference**
Myocardial fibrosis	Pro-fibrosis	199b	Up	Dyrk1a	PMID:21102440
		154	Up	–	PMID:26928825
		21	Up	TGFβR1	PMID:29808534
		223	Up	RASA1	PMID:29689569
		181	Down	–	PMID:32538237
		30	Down	–	PMID:32418505
		30	Down	PTAFR	PMID:32329883
		154	Down	GSK-3β	PMID:29687862
		433	Down	TGFβR1	PMID:27698941
		378	Down	TGFβR1	PMID:25104350
		378	Down	MKK6/p38	PMID:29721099
		22	Down	TGFβR1	PMID:27997889
	Anti-fibrosis	206	Up	TiMP3	PMID:21731608
		132	Up	PTEN	PMID:30216493
		146	Up	EGR1	PMID:30362610
		133	Up	TGFβR1	PMID:31646592
		9-5p	Down	Fstl1	PMID:30101604

*miRNAs are divided into pro-fibrosis and anti-fibrosis according to functions. The pro-fibrosis of main targets include TGFβR1 and the anti-fibrosis of main targets include TGFβR1 and PTEN*.

## Cardiomyocyte Hypertrophy

Cardiac hypertrophy is an adaptive response when the heart faces various pathological stimuli, such as energy metabolism disorders, increased load, changes in humoral factors, and neuroendocrine activation (82, 83). With myocardial contractility decreasing after MI, ventricular remodeling always occurs with compensatory hypertrophy of the myocardium (84). Although this mechanism has an important role for cardiac function in the early phase of MI, these changes will eventually develop into heart failure and even death (85). Myocardial contractility depends mainly on the expression of two myocardial myosin heavy chain (MHC) genes α and β, called Myh6 and Myh7, respectively (86). Thyroid hormone T3 signaling controls the expression of these two MHC genes by stimulating the expression of Myh6 and inhibiting the expression of Myh7 after birth (87). miR-208 is a heart- and muscle-enriched miRNA (42). Transgenic overexpression of miR-208a in the heart, which is encoded within an intron of Myh7 and regulates the thyroid hormone-associated protein 1 (TRβ1), was shown to be sufficient to induce hypertrophic growth of the heart in mice (88). Another study showed that infarcted hearts have a higher abundance of extracellular vesicular miRNA-27a compared with normal hearts, and that miRNA-27a inhibited PDZ and LIM domain 5 (PDLIM5) translation, leading to cardiomyocyte hypertrophic gene expression (89). Probably, Myh7 is also regulated by the T-box transcription factor 5 (Tbx5) (90). Mice injected with an adeno-associated virus expressing miR-1 showed reduced, and even reversed, myocardial hypertrophy (91). miR-1 is a heart- and muscle-enriched miRNA (42). miR-1 inhibits the expression of cell division protein kinase 6 (CDK6) to inhibit phenylephrine-induced neonatal rat ventricular cardiomyocytes hypertrophy, thereby attenuating the inhibition of the expression of β-MHC and phosphorylated the retinoblastoma protein (92). miR-340 is a pro-eccentric hypertrophy miRNA that targets the cardiomyocyte structure protein dystrophin (93). miR-22 and miR-495 have the opposite effect, with their upregulation significantly increasing cell size and markedly decreasing the expression of Myh6 (94). Moreover, they negatively regulate the PTEN levels in cardiomyocytes (95). Additionally, overexpression of let-7a was found to repress the expression of ANP, BNP, and Myh7, as well as of CaM levels (96). A dual-luciferase reporter assay also showed that let-7a could bind to the 3'–UTR of CAM1 and let-7a possesses a prominent anti-hypertrophic property by targeting CAM genes (97).

There is a potential link between cardiac hypertrophy and cardiac cell death (98, 99). A study suggested that intravenous miR-144 has a potent effects on cardiac remodeling of rats with MI, which was associated with significant changes in autophagy signaling (100). Cy3-labeled miR-144 was localized to the infarct and border zones and was taken up by cardiomyocytes and macrophages (101). Similarly, knock-down of the autophagy-related protein 9 (ATG9A), which is a direct target of miR-34, downregulated the autophagic activity and cardiomyocyte hypertrophy (102). Furthermore, overexpression of the S-phase kinase-associated protein 2 (Skp2) promoted autophagy and rescued cardiac hypertrophy induced by Ang II. And, Skp2 knock-down further inhibited autophagy and cardiac hypertrophy in mice with MI (103). In contrast, increased miR-206 expression induced cardiac hypertrophy and inhibited cell death in cultured cardiomyocytes. The Yes-associated protein can promote cardiomyocyte growth and survival in postnatal hearts, and increases the abundance of miR-206, which in turn plays an essential role in mediating hypertrophy and survival by silencing FOXP1 in cardiomyocytes (39). miR-133, 541, 200, 624, and 181 can in turn inhibit hypertrophy and improve cardiac function through different mechanisms (104, 105). Recently, a study confirmed that upregulated miR-142-3p could inhibit hypertrophy and mitochondrial SH2B1, a key factor regulating energy metabolism (106). Moreover, miR-195-5p and miRNA-20a-5p can promote cardiac hypertrophy via targeting mitofusin-2 (MFN2), which is a mitochondrial outer membrane fusion protein (107, 108). TRβ1/Myh7, Ang II, and PTEN have been the main targets of research, and MFN2 may be a new major target (Table 3).

**Table 3:** Micro RNAs targets and functions in cardiomyocyte hypertrophy.

	**Functions**	**miRNA**	**Up/down**	**Targets**	**Reference**
Cardiomyocyte hypertrophy	Pro-hypertrophy	208	Up	Trβ1/MYH7	PMID:19726871
		27	Up	Trβ1/MYH7	PMID:21149577
		27	Up	PDLIM5	PMID:32370947
		212/132	Up	Ang II	PMID:23011132
		22	Up	SIRT	PMID:23524588
		22	Up	PTEN	PMID:21618527
		206	Up	FoXP1	PMID:26333362
		340	Up	DMD1	PMID:26084457
		365	Up	SKP2	PMID:28130111
		195	Up	MFN2	PMID:31341888
		20	Up	MFN2	PMID:31295012
		10	Down	TNX5	PMID:28100873
		200	Down	MLCK	PMID:30680929
		34	Down	Ang II	PMID:24728149
		1	Down	CDK6/NFAT	PMID:26699910
		133a	Down	RhoA	PMID:17468766
	Anti-hypertrophy	1	Up	FbIn2	PMID:23612897
		541	Up	MITF	PMID:24722296
		let-7	Up	CaM	PMID:28123343
		142	Up	SH2B1	PMID:30372837
		672	Up	JUN	PMID:29339068
		144	Up	MOTR	PMID:30084039
		181	Down	–	PMID:32538237
		410/495	Down	–	PMID:26999812
		495	Down	PTEN	PMID:29566365

*miRNAs are divided into pro-hypertrophy and anti-hypertrophy according to functions. The pro-hypertrophy of main targets include Trβ1/MYH7, MFN2, and Ang II and the anti-hypertrophy of main targets include MOTR, PTEN, and CaM*.

## Therapeutic Targets

The majority of patients who survive MI experience a loss of functional cardiomyocytes as a result of the ischemic injury, which leads to ventricular failure with significant alteration of the quality of life and increased risk of mortality (109). Since the proliferation and self-healing capacity of cardiomyocytes in adults is limited, regeneration therapy has emerged as an attractive concept for cardiac repair (110). Compared with traditional interventional stent reperfusion, regenerative therapy can save the myocardium or even regenerate it by promoting angiogenesis, and inhibit, or even avoid adverse cardiac remodeling (111). The main directions of regenerative therapy include stem cell therapy, cardiac fibroblast reprogramming, and exosome therapy.

Scholars have focused on the development of induced pluripotent stem cells, but such treatments have failed to achieve significant benefits in clinical trials (112). This approach has demonstrated limited therapeutic effect mainly due to the risk of immune rejection, genetic instability, tumorigenic potential, low induction efficiency (in the case of induced pluripotent stem cells), and ethical issues (in the case of embryonic stem cells use) (113–115). Leda et al. successfully reprogramed mouse heart and skin fibroblasts into functional induced cardiomyocytes (iCMs) *in vitro* (19). However, the cardiac fibroblast reprogramming efficiency was extremely low and its requirements are too draconian (116). Furthermore, the iCMs carry other risks such as arrhythmias (117). However, the emergence of exosomes provided an additional tool for myocardial regeneration. Exosomes started to attract attention in 2007, when it was discovered that they have the unique property of transferring miRNAs between cells *in vivo*, acting as miRNA nanocarriers (118). Recently, mounting evidence has demonstrated the potential of stem cell-derived exosomes, as well as other exosome types, in repairing damage after MI (62, 119). A study confirmed that mesenchymal stem cell (MSC)-derived exosomes electroporated with miR-132 mimics could markedly enhanced the neovascularization in the peri-infarct zone and preserve heart functions (120). Additionally, an injection of exosomes over-expressing miR-21 directly into the infarct zone was found to markedly inhibit cell apoptosis and significantly improve cardiac function in mice (121). MSC-derived exosomes were also found to protect the heart in a porcine model of MI when administered systemically by intravenous injection (122). However, major hurdles remain for the use of exosomes, primarily due to low yields from cell cultures coupled with complicated purification processes (123). Nevertheless, a study reported the self-assembly of a stem cell membrane-camouflaged exosome-mimicking nanocomplex that recapitulated exosome functions, achieving efficient miRNAs delivery and miRNA-mediated myocardial repair (22). Furthermore, a group constructed a functionalized single-walled carbon nanotube bound to siRNA from caspase 3 (F-CNT-siCas3) that demonstrated good water solubility and biocompatibility, but also had a high transfection efficiency of up to 82%, significantly downregulating the expression of the caspase 3 gene and protein *in vivo* (124). A low molecular weight heparin-encapsulated exosome nanocomplex demonstrated that it could overcome a microvascular obstruction in the infarct, and this structure not only makes myocardial cells uptake miRNAs, which will promote cardiac repair, but will also prevent myocardiocyte apoptosis and attenuate myocardial fibrosis (125). Although the exosome nanocomplex technology is expensive and holds uncertain side effects, it greatly improves the cell conversion rate compared with the previous two regeneration methods, while showing good *in vivo* results. Thus, an exosome nanocomplex is conducive to further clinical research.

## Conclusion

In particular, miRNAs play an important role in the pathology of myocardial apoptosis, fibrosis, and hypertrophy after MI. Targets of miRNAs have significant therapeutic potential, although there are still some conflicting data. The majority of miRNAs and their targets have consistent actions. In particular, SIRT, Bcl-2, Bax, caspase, TGF-β1, TRβ1/Myh7, and MFN2 are believed to play a more significantly prominent role than other targets. In addition, with the development of exosome therapy in combination with nanomaterials, some of the limitations of stem cell therapy (such as low conversion rates and poor cardiac absorption) can be overcome. Exosome nanocomplexes cannot only carry myocardium-friendly miRNAs, but can also directly deliver analogs of important targets into the myocardium in the future. Whether exosome nanocomplexes can treat infarcted myocardium by acting as vectors for the main targets of miRNAs, similar to cocktail therapy, may be the next major direction of exploration. Exosome nanocomplexes with miRNAs are more likely to be successfully taken forward into clinical evaluation than other experimental strategies; however, they also have several limitations. First, the up- and down-stream relationship with the target needs further verification and improvement. There are still conflicting effects of miRNAs (such as miR-Let-7 and miR-154) and more experimental studies are needed. Second, miRNAs act on multiple targets and are involved in several mechanisms; thus, it is necessary to weigh the advantages and disadvantages of their activities. Lastly, treatment with miRNAs is complicated and expensive, and more clinical studies are needed to confirm their therapeutic potential. With the perfectly targeted mechanism and the continuous improvement of exosome therapeutic materials, we believe that mature technologies and drugs based on miRNAs used to save the infarcted myocardium will soon be available to all.

## Author Contributions

HZ supervised the writing of the manuscript. WW and HZ prepared the manuscript and wrote the draft together. WW prepared the figures. All authors have read and agreed to the published version of the manuscript.

## Conflict of Interest

The authors declare that the research was conducted in the absence of any commercial or financial relationships that could be construed as a potential conflict of interest.
